# Role of IQ Motif-Containing GTPase-Activating Proteins in Hepatocellular Carcinoma

**DOI:** 10.3389/fonc.2022.920652

**Published:** 2022-06-16

**Authors:** Qingqing Dai, Quratul Ain, Michael Rooney, Fei Song, Alexander Zipprich

**Affiliations:** ^1^ Department of Internal Medicine IV (Gastroenterology, Hepatology, and Infectious Diseases), Jena University Hospital, Jena, Germany; ^2^ Else Kröner Graduate School for Medical Students “Jena School for Ageing Medicine (JSAM)”, Jena University Hospital, Jena, Germany; ^3^ Department of Urology, Jena University Hospital, Jena, Germany

**Keywords:** IQGAPs, hepatocellular carcinoma, HBV, cytoskeleton, signal transduction

## Abstract

IQ motif-containing GTPase-activating proteins (IQGAPs) are a class of scaffolding proteins, including IQGAP1, IQGAP2, and IQGAP3, which govern multiple cellular activities by facilitating cytoskeletal remodeling and cellular signal transduction. The role of IQGAPs in cancer initiation and progression has received increasing attention in recent years, especially in hepatocellular carcinoma (HCC), where the aberrant expression of IQGAPs is closely related to patient prognosis. IQGAP1 and 3 are upregulated and are considered oncogenes in HCC, while IQGAP2 is downregulated and functions as a tumor suppressor. This review details the three IQGAP isoforms and their respective structures. The expression and role of each protein in different liver diseases and mainly in HCC, as well as the underlying mechanisms, are also presented. This review also provides a reference for further studies on IQGAPs in HCC.

## 1 Introduction

IQ motif-containing GTPase-activating proteins (IQGAPs) are a family of evolutionarily conserved proteins found in eukaryotic cells, ranging from yeast to humans, and consist of three closely related homologs; IQGAP1, IQGAP2, and IQGAP3. These homologs comprise multiple domains involved in regulating a broad spectrum of biological processes such as cytoskeleton remodeling, cytokinesis, protein trafficking, cell adhesion, proliferation, migration, and tumorigenesis (1–6). Aberrant IQGAP expression has been extensively reported in a range of malignancies, including liver, gastric, lung, breast, and colon cancers, and is highly related to poor clinical characteristics and prognosis (7–10).

Globally, liver cancer is a significant public health threat with increasing prevalence. By 2025, liver cancer is anticipated to affect more than one million individuals annually (11). Hepatocellular carcinoma (HCC), the most common subtype of primary liver cancer, which is the sixth most frequently diagnosed type of cancer and the third most common cause of cancer-related mortality worldwide in 2020 (12). Several imperative risk factors such as chronic Hepatitis B and C virus (HBV and HCV) infections, alcoholic and non-alcoholic liver diseases, e.g., non-alcoholic fatty liver disease (NAFLD) and the advanced state of NAFLD termed as non-alcoholic steatohepatitis (NASH), and hereditary hemochromatosis have been described for HCC (13). Apart from these risk factors, any other causes leading to liver cirrhosis, including HBV and HCV, are also identified as crucial risk factors for HCC (11, 14). Over the last decade, the discovery of novel biomarkers and innovation in imaging technologies have led to tremendous progress in the management and therapy of HCC. Hepatectomy and liver transplantation have been the mainstays of treatment for patients with HCC (15). Additionally, alternative adjuvant therapies such as radiofrequency ablation, transarterial chemoembolization, and immunotherapy have dramatically improved patient prognosis (11).

IQGAPs are important molecular factors that play a comprehensive and distinctive role in HCC initiation and progression. IQGAP1 is overexpressed in HCC and contributes to cancer development and advancement (16, 17). IQGAPs have complex interactions with several different factors at the molecular level and thus play a vital role in cancer progression. Likewise, IQGAP1 can bind to the cell cycle regulators and might boost cell division and enhance the invasive and migratory abilities of HCC cells, as shown for the Huh-7 cell line (18). Moreover, IQGAP1 also interacts with certain transcription factors and co-activators and affects the signaling pathways involved in apoptosis and survival processes (19). Additionally, IQGAP1 has been shown to render anoikis resistance and metastasis in HCC (19, 20). Similar to IQGAP1, IQGAP3 is considered an oncogene in HCC, where it supports intrahepatic and distant metastasis and epithelial-to-mesenchymal transition (EMT) (21, 22). In contrast to isoform 1 and 3 of IQGAP, IQGAP2 is considered a tumor suppressor in HCC since its expression coincides with patient prognosis and is decreased in HCC tissues (23). Furthermore, IQGAP2-deficient mice were more prone to spontaneously growing HCC (24). Therefore, all these findings indicate that IQGAPs are potential therapeutic targets in HCC. This review details the three IQGAP isoforms and their distinct structures. In addition, we compare the characteristics of IQGAPs and summarize their expression and functions in different liver diseases and mainly in HCC, as well as their associated mechanisms.

## 2 The Isoforms of IQGAPS

Although IQGAPs share a similar domain composition, they differ in tissue expression, subcellular localization, and function. IQGAP1, the most extensively investigated isoform among IQGAPs, has gained considerable attention since its discovery in 1994 (25). IQGAP1 is a ubiquitously expressed scaffold protein involved in various vital cellular functions (4, 5). IQGAP1 has been postulated to maintain matrix homeostasis *via* collagen phagocytosis in physiological tissue remodeling (26). Furthermore, IQGAP1 appears to be an exceptionally attractive therapeutic target because it acts as a hub for signaling pathways involved in cancer progression (20, 27–29).

IQGAP2 was initially identified in 1996 as a large cytoplasmic scaffold protein that is predominantly expressed in the liver but also in the prostate, kidney, stomach, testis, and platelets (30). IQGAP2 is a tumor suppressor, as a decreased expression has been observed in human breast cancer and HCC (23, 31). Moreover, IQGAP2-mutant mice are susceptible to developing HCC. Additionally, IQGAP2 inhibits EMT and angiogenesis and promotes apoptosis in breast cancer. Thereby, low expression levels of IQGAP2 results in a poor prognosis for patients (7, 31).

IQGAP3 has received scant attention despite its discovery in 2007 (32) and its detection in various organs, including the liver, stomach, ovaries, prostate, breast, pancreas, and lung (8, 33–38). IQGAP3 is mainly expressed in brain (39) and is critical for regulation of neurite outgrowth of the neurons (32). In addition to regulating mitosis progression, genome integrity, and stability, IQGAP3 is necessary and sufficient for proper cell proliferation and migration (35, 40). Accordingly, IQGAP3 is classified as an oncogene owing to its intimate association with tumorigenesis and metastasis. Its expression is inversely linked with clinical characteristics and survival in most cancers (8, 38, 41, 42).

## 3 The Molecular Domains of IQGAPS

**Figure 1:**
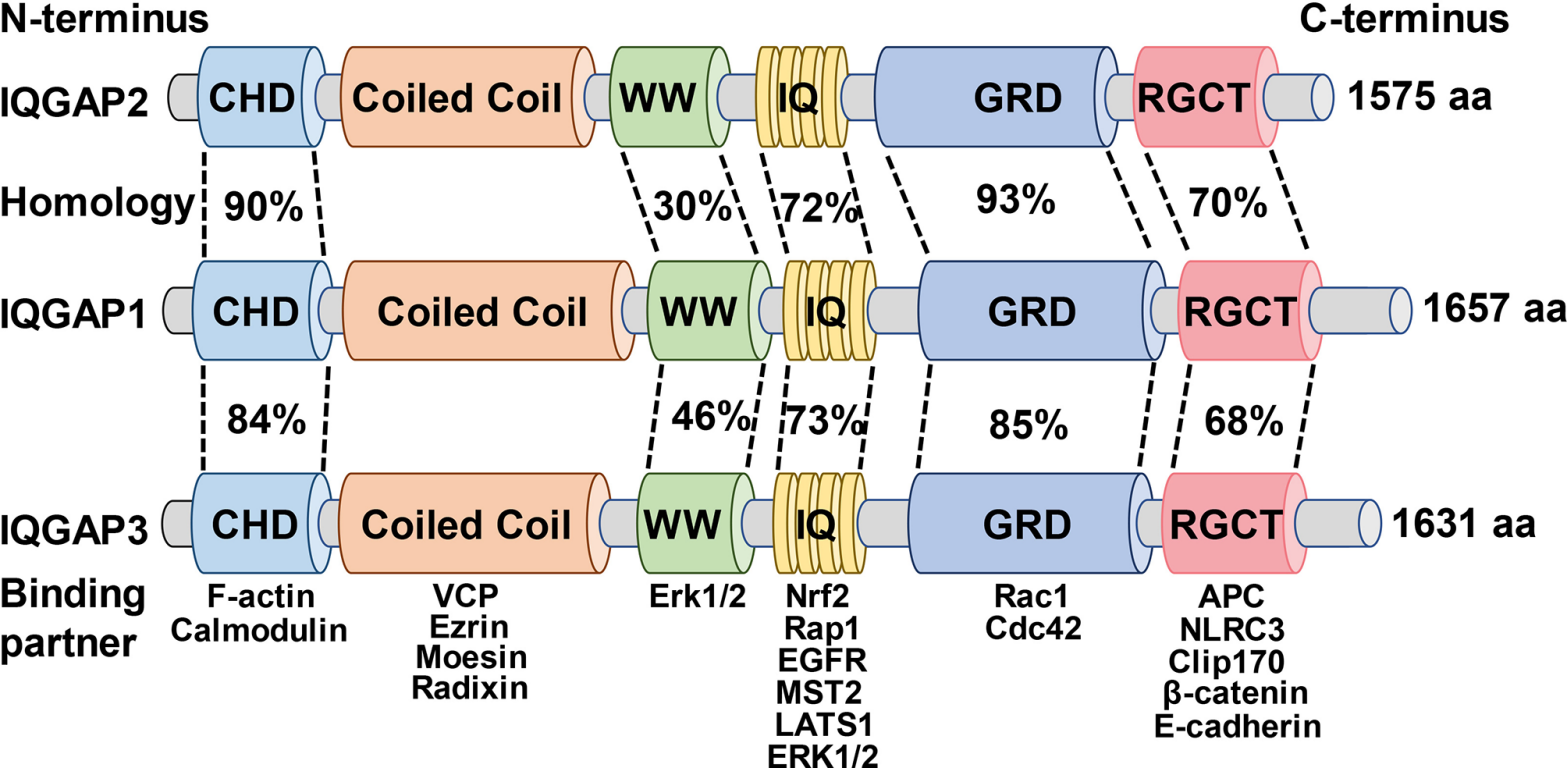
Schematic illustration of the domain organization and amino acid homology of the IQGAPs family and the binding partners of the respective domains.

IQGAPs have a high degree of amino acid sequence homology and a comparable domain structure (**Figure 1**), with IQGAP1 having 62% and 59% amino acid sequence identity to IQGAP2 and IQGAP3, respectively (43, 44). IQGAPs contain six distinct functional domains depending on the amino acid sequence. These diverse domains allow them to bind to a variety of partners and modulate the spatiotemporal distribution of various signal transduction complexes (9, 45).

Starting from the N-terminus, IQGAPs comprise a calmodulin homology domain (CHD) that mediates actin-binding (46). For instance, the interaction between the CHD of IQGAP1 and actin filaments governs the cytoskeleton modulation to facilitate actin binding and polymerization, thus further regulating cell division, cell migration, and the stability of cell-cell contacts (47). Moreover, it has been shown previously that IQGAP1 dynamically contributes to the formation of the spine head in the cultured rat hippocampal neurons through CHD (48).

Following the CHD is the coiled-coil (CC), also known as heptad domains, which are characterized by the presence of repetitive hydrophobic and charged amino acids (49). The CC repeat sequence enables IQGAP1 and IQGAP2 to bind to the ERM protein family (ezrin, radixin, and moesin) that crosslinks actin-based cytoskeletons to plasma membranes and participates in multiple intracellular signaling pathways, reinforcing the role of IQGAPs as cytoskeletal regulators and signal transduction hubs (50, 51). Additionally, the CC repeat region of all IQGAPs isoforms stimulates neural development and spine morphogenesis *via* interaction with valosin containing protein (VCP) which also suggests a crucial role of IQGAPs in the pathophysiology of neurodegenerative diseases (52).

The polyproline protein-protein (WW) domain contains two functionally conserved tryptophans, W, which interacts with other proteins, including proline-rich regions. WW domain of IQGAP1, through a polyproline motif, binds to classical MAP kinases (MAPK) and activates downstream signaling pathways and advances tumor development, growth, and invasion (53, 54). Likewise, interruption of IQGAP1-kinase- connections by ectopic expression of WW peptide in mice selectively suppressed Ras-MAPK -mediated oncogenesis and tumor invasion. Moreover, this effect also remarkably improves the life span of tumor-bearing mice (54). However, a later study reported contradictory findings that the WW domain of IQGAP1 was neither sufficient nor essential for binding to MAPK, whereas the IQ domain that immediately follows WW domain was both adequate and necessary for high-affinity IQGAP1-kinase interaction (55).

IQ domain comprises of four tandem isoleucine-glutamine (IQ) motifs. This domain interacts with calmodulin, a calcium-sensing protein capable of binding to and regulating a diverse array of target proteins (56). The IQ domain also interacts with protein kinases and cell surface receptors such as epidermal growth factor receptor (EGFR) (55, 57–60). The IQ domain of IQGAP1 activates MAPK signaling cascade by binding to EGFR, which is associated with epidermal cancer progression and invasion (61). Subsequently, IQGAP1-IQ motif decoy peptide supplementation *in vivo* inhibited the oncogenic signaling pathway and carcinogenesis without compromising normal epidermal proliferation or differentiation (61).

The IQ domain is followed by a GTPase activation-related structural domain (GRD) that is responsible for the nomenclature of IQGAPs (49). This domain is highly homologous to the functional components of Ras GTPase-activating proteins (GAPs) and interacts with small GTPases (3, 9, 62). The crystal structure of GRD showed that threonine replaces the catalytic “arginine finger” required for GTP hydrolysis; thus, it does not have GAP function, failing to hydrolyze GTP but rather stabilizing the GTP-bound protein in its active state (63). Such interaction between GRD domain of IQGAP2 and small GTPases is mechanistically crucial for the polymerization of actin filaments during cancer progression (64, 65).

Finally, IQGAPs contain a RasGAP C-terminal (RGCT) structural domain that significantly contributes to cell-cell adhesion, cell polarization, and directional migration by binding to a variety of proteins, including E-cadherin and β-catenin (66–69). Furthermore, IQGAP1 enhanced extracellular matrix (ECM) degradation by binding to the exocyst subunits *via* RGCT domain to prime tumor cell metastasis and invasion, while the elimination of the exocyst binding site abrogated the enhancement of IQGAP1-induced ECM degradation (70, 71). This indicates that IQGAP1-regulated cytoskeletal remodeling plays an aggressive role in tumor progression. Likewise, mutations of the RGCT can severely impair the interaction of IQGAP1 with small GTPases; therefore, complying that the domains adjacent to GRD are also crucial for the binding of IQGAPs with small GTPases of Rho family proteins (72–75).

## 4 The Relationship Between IQGAPS and Liver Diseases

As delineated above, IQGAP scaffold proteins are crucial for a variety of vital cellular processes, and their molecular interactions with other biological molecules are very important for the development and progression of cancer. Likewise, role of IQGAPs has been quite extensively explored in various cancers including HCC. However, the investigations for the implications of IQGAPs in other liver diseases such as fatty liver disease, fibrosis, and cirrhosis are still too scarce and need more scientific research to explore the role and importance of these scaffold proteins in liver diseases (76–79).

### 4.1 Fatty Liver Disease

Fatty liver disease is a chronic liver disease also worthy of attention, affecting more than 25% of the population globally (80). Moreover, studies have shown that IQGAPs play an essential role in fatty acid and glucose metabolism (76–78). Likewise, a recent study demonstrated that in IQGAP1 knock out mice (*Iqgap1^–/–^*), fatty acid oxidation and ketogenesis were significantly reduced compared to the wild type (WT) counterparts. Moreover, livers from *Iqgap1^–/–^* mice showed pale appearance and exhibited microsteatosis under a high-fat diet, concomitant with elevated levels of hepatic triglyceride than WT mice implying the compromised roles of peroxisome proliferator-activated receptor alpha (PPAR-α) and mammalian target of rapamycin (mTOR), essential regulators of fatty acid β-oxidation in livers of *Iqgap1^–/–^* mice (76). In addition, a previous study by Chawla and colleagues showed that IQGAP1 null mice exhibited impaired glucose tolerance and were unable to clear glucose as efficiently as WT mice. The defective glucose homeostasis might be due to impaired insulin signaling in the absence of IQGAP1 (77). These data imply that IQGAP1 plays a significant role in the complex metabolic regulatory pathways, especially that of fatty acid, glycogen, and insulin signaling in the liver (76, 77). Likewise, the role of IQGAP2 in metabolic homeostasis was demonstrated by Vaitheesvaran and colleagues. IQGAP2 null mice fed with a standard laboratory diet displayed impaired glucose and fatty acid metabolism, suggesting that IQGAP2 deletion results in a pre-diabetic hepatic environment paving the way for the NAFLD manifestation. Moreover, *Iqgap2^-/-^* mice had high blood glucose levels and were overweight compared to the WT mice (81). However, another previous study showed that abrogation of IQGAP2 protects against diet-induced hepatic steatosis due to compromised uptake of fatty acid. Hepatocytes from *Iqgap2^-/-^* mice fed with high-fat diet displayed selective loss of the facilitated phase of long-chain fatty acids (LCFA) uptake and retention of the intact passive phase of LCFA uptake. Additionally, *Iqgap2^-/-^* livers expressed remarkably lower *de novo* synthesis of lipids in the hepatocytes than the WT counterparts. Similarly, livers from IQGAP2 null mice depicted improved sensitivity to insulin. These findings suggest the fundamental role of IQGAP2 in the coordination of metabolic physiological processes involved in the cellular uptake of functional fatty acid and lipid, lipid synthesis and processing, and, plausibly, regulating glucose levels (78). Apart from the delineated roles of IQGAP1 and IQGAP2, additional research is required to further explore the role of IQGAPs in the pathophysiology of fatty liver disease.

### 4.2 Fibrosis

Fibrotic liver occurs in most types of chronic liver ailments, e.g., chronic HBV and HCV infections and alcoholic fatty liver disease, and is characterized by the excessive buildup of ECM proteins including collagen. Fibrosis is an aberrant tissue restoration process in response to long-term and persistent liver injuries that may further develop into liver cirrhosis, failure, or cancer if left untreated. Hitherto, chronic liver diseases coupled with fibrotic liver have resulted in remarkable worldwide morbidity and mortality (82–84). Since IQGAP1 is one of the vital scaffolding proteins that have established pivotal roles in cytoskeleton remodeling and rearrangements of cellular networks and various vital cellular processes. It implies that IQGAP1 might have a potential role in the modulation of liver fibrosis. Likewise, in a recent study, mRNA and protein levels of IQGAP1 were shown to be significantly elevated in CCl_4_-induced liver fibrosis mice and TNF-α-treated hepatic stellate cell (HSC) line, LX-2 cells, which might be related to the development and advancement of liver fibrosis. Furthermore, the overexpression of IQGAP1 in activated LX-2 cells promoted the secretion of inflammatory cytokines. These findings suggest the putative role of IQGAP1 in liver inflammation and fibrogenesis (85). The increased levels of IQGAP1 in fibrotic liver were also confirmed by Ma and colleagues, who further demonstrated the cell type fraction responsible for such increase (79). IQGAP1 expression was found to be remarkably elevated in non-parenchymal cells and myofibroblasts during murine liver fibrosis (79). Sphingosine 1-phosphate (S1P) mediated bone marrow mesenchymal stromal cells (BMSCs) migration from bone marrow to the injured liver is one of the primary sources of myofibroblasts (86). Likewise, Ma and colleagues showed that IQGAP1 mediated SIP-induced BMSC migration and motility (79, 86). S1P induces membrane translocation of IQGAP1 and promotes the interaction between IQGAP1 and Cdc42/Rac1 to facilitate BMSC migration to the liver. Moreover, the knockdown of IQGAP1 significantly reduced cell viability and migratory capacity in BMSC cells (79).

### 4.3 Viral Hepatitis

Viral infections of liver, especially HBV and HCV infections, are one of the top infectious diseases and are responsible for the demise of 1.4 million people per annum worldwide (87, 88). IQGAP2, mainly expressed in the liver, is shown to have a novel role in the innate antiviral response. The functional analysis revealed that IQGAP2 regulates many of the interferon (IFN) required anti-hepatitis C viral genes together with RelA subunit of nuclear factor κB (NF-κB). Following IFN treatment, IQGAP2 physically interacts with RelA to mediate the early activation of RelA and the downstream targets of NF-κB, which ultimately protects against HCV infection (89). Several studies have also suggested the involvement of IQGAPs in HBV-induced HCC, as detailed in the section “Action mechanisms of IQGAPs in HBV-caused HCC”.

### 4.4 Hepatocellular Carcinoma

IQGAPs have attracted increasing interest in cancer research because of their fundamental contribution to cytoskeletal remodeling and signal transduction. Numerous studies have shown an association between IQGAPs and HCC initiation, metastasis, and recurrence, as well as poor prognosis in patients with HCC (10, 16–24, 29, 81, 90–98) (details in **Table 1**).

**Table 1:** Summary of the role of IQGAPs in tumor oncogenesis and related function/clinical characteristics.

Year(refs)	Species^*^	Pathway	Function/Clinical characteristics of IQGAPs	
2008 (24)	M	Wnt-β-catenin-cyclin D1	IQGAP1	hepatocarcinogenesis↑
			IQGAP2	cancer initiation↓
2008 (90)	H	–	IQGAP3	hepatocarcinogenesis↑
2010 (17)	H/M/C	IQGAP1-PI3K-AKT	IQGAP1	cell proliferation↑
2010 (91)	H/C	–	IQGAP1	hepatocarcinogenesis↑
			IQGAP2	cancer initiation↓
2013 (92)	H/M/C	Bile acid-IQGAP1-YAP1	IQGAP1	cell proliferation↑; cellular adhesion↓; hepatocarcinogenesis↑
2013 (93)	H/M	Wnt-β-catenin	IQGAP2	cancer initiation↓
2014 (81)	M	–	IQGAP2	maintain redox equilibrium; cell proliferation↓
2014 (23)	H	–	IQGAP1	tumor size↑; TNM stage↑; tumor differentiation↓; disease-free survival↓; overall survival time↓
			IQGAP2	tumor size↓; TNM stage↓; tumor differentiation↑; disease-free survival↑; overall survival time↑
2015 (94)	H/C	IQGAP1-β-catenin	IQGAP1	tumor differentiation↓; cell proliferation↑; migration↑
2015 (95)	M	IQGAP1-Ras	IQGAP1	hepatocarcinogenesis↑
2016 (96)	H	–	IQGAP3	tumor size↑; intrahepatic metastasis↑; HBsAg↑; TNM stage↑
2016 (97)	C	IQGAP1-Ras	IQGAP1	cell growth↑; proliferation↑; invasion↑; apoptosis↓
2017 (16)	H/C	–	IQGAP1	intrahepatic metastasis↑; microvascular invasion↑; tumor recurrence risk↑; mortality↑; cell migration↑; invasion↑; EMT↑
2017 (22)	H/M/C	IQGAP3-TGF-β	IQGAP3	cell migration↑; invasion↑; EMT↑; TNM stage↑; tumor size↑; intrahepatic metastasis↑; overall survival↓
2017 (18)	C	HBx-CDC42-IQGAP1	IQGAP1	cell proliferation↑; migration↑; apoptosis↓
2019 (21)	H/M/C	E2F1-IQGAP3-PKCα-PI3K-AKT	IQGAP3	tumor size↑; HBsAg↑; intrahepatic and distant metastasis↑; overall survival time↓; cell proliferation↑
2020 (20)	H/M/C	HBV-ROS-IQGAP1-Rac1-Src/FAK	IQGAP1	tumor size↑; HBsAg↑; AFP↑; intrahepatic metastasis↑; BCLC stage↑; overall survival↓; cell migration↑; invasion↑; anoikis↓
2021 (98)	C	AMD1-IQGAP1-FTO-NANGO/SOX2/KLF4	IQGAP1	stemness↑
2021 (29)	M/C	IQGAP1- YAP1- NUAK2	IQGAP1	cell proliferation↑; tumor growth↑
2021 (19)	C	IQGAP1-MST2/LATS1-YAP1;IQGAP1-AKT/ERK	IQGAP1	cell proliferation↑; cell apoptosis↓; tumorigenesis↑
2022 (10)	M	IQGAP1/NF-kB	IQGAP1	hepatocarcinogenesis↑

*H, human; M, mice; C, cell. The symbol “↑, ↓” mean increase and decrease, respectively.

IQGAP1 is considered an oncogene in HCC as its mRNA and protein levels are elevated in human HCC tissues compared to para-tumor and normal liver tissues. Moreover, high IQGAP1 expression is associated with poor clinical outcomes (16, 17, 23, 91). In HCC tissues, IQGAP1 expression is positively correlated with tumor size, number, stage, and HBV surface antigen (HBsAg) and alpha-fetoprotein (AFP) expression but inversely correlated with tumor differentiation (16, 23). IQGAP1 promotes microvascular invasion and distant metastasis in HCC, accounting for the higher postoperative recurrence rate and shorter disease-free survival and overall survival of patients with elevated IQGAP1 expression (16). IQGAP1 expression is also upregulated in HBV-induced HCC and is linked to poor prognosis (20).

Consistent with the predominant expression of IQGAP2 in the liver, convincing evidence supports a tumor suppressor role for IQGAP2 in HCC. Decreased IQGAP2 expression is observed in most patients with HCC and is associated with larger tumor size, advanced tumor stage, and poorer tumor differentiation, as well as shorter postoperative tumor-free survival and overall survival after hepatectomy (23, 91). Both low IQGAP2 and high IQGAP1 levels have been reported as independent prognostic risk factors for poor postoperative survival in patients with HCC. Furthermore, patients with IQGAP1^+^/IQGAP2^-^ tumors have the worst prognosis, whereas those with IQGAP1^-^/IQGAP2^+^ tumors have the best prognosis (23). Thus, positive IQGAP1 and negative IQGAP2 are substantially related to HCC progression, validating the distinct functions of IQGAP1 and IQGAP2 in HCC.

IQGAP3, like IQGAP1, is also considered an oncogene in HCC. IQGAP3 is overexpressed in HCC tissues. Its expression is associated with larger tumor size, advanced tumor stage, and poor tumor differentiation (22, 96). IQGAP3 promotes intrahepatic and extrahepatic metastasis in HCC, which dramatically shortens patient survival time (21, 22). Furthermore, IQGAP3 is a novel biomarker for HCC screening and diagnosis and is superior to AFP for detecting small HCCs (96). IQGAP3 can be used as a complementary biomarker of AFP to improve the accuracy of AFP-negative HCC. The combination of AFP, IQGAP3, and chaperonin containing TCP1 complex subunit 3 (CCT3) significantly enhanced the discriminatory ability of HCC compared to AFP alone (96).

## 5 Action Mechanism of IQGAPS in HCC

**Figure 2:**
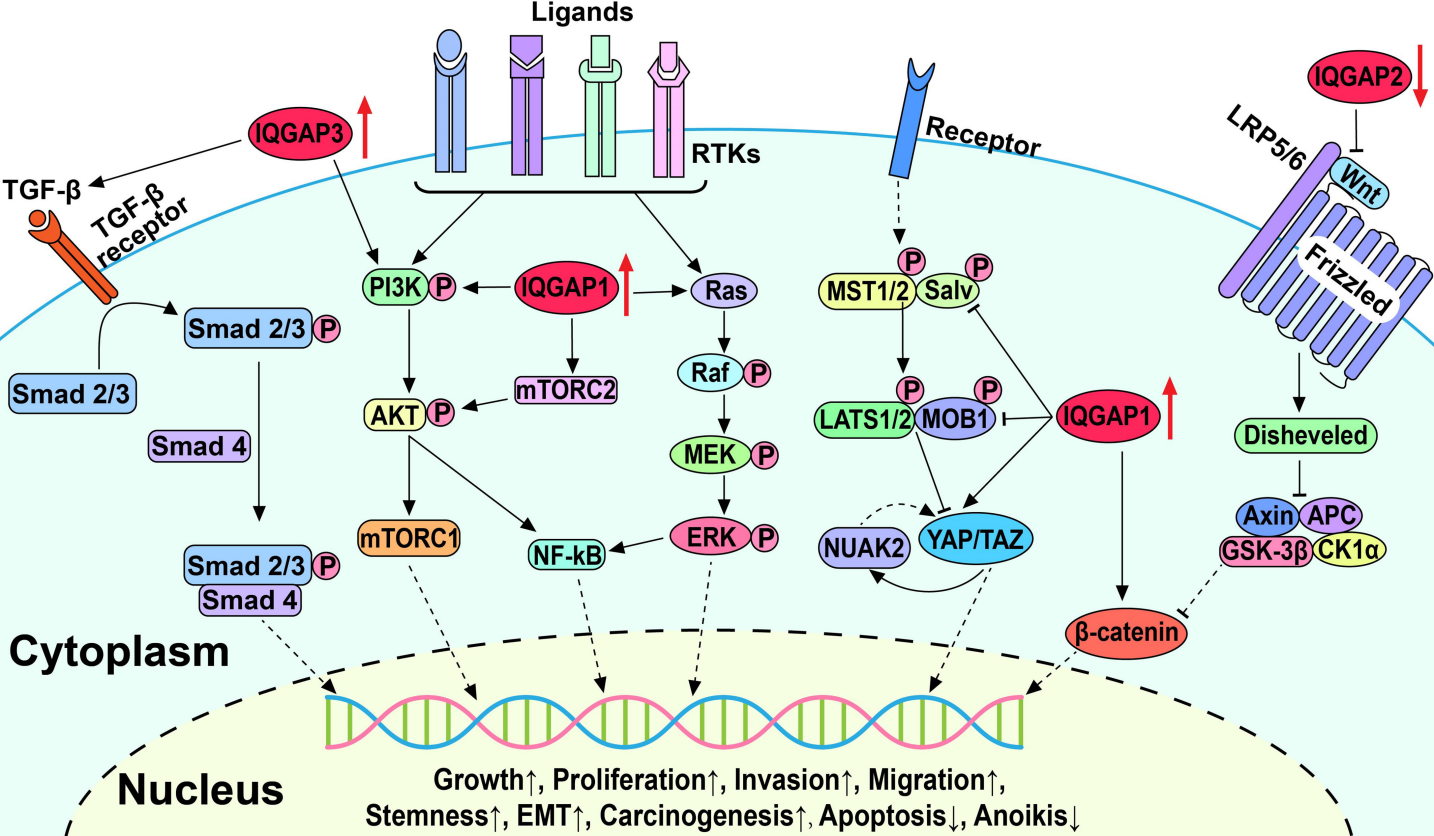
Schematic diagram of IQGAPs-mediated signaling pathways involved in the pathogenesis of hepatocellular carcinoma.

Aberrant activation of multiple signaling pathways in HCC, such as the Wnt/β-catenin, Hippo, transforming growth factor β (TGF-β), receptor tyrosine kinase (RTK)-activated phosphatidylinositol 3-kinase/AKT/mTOR (PI3K/AKT/mTOR), and Ras/Raf/MEK/ERK (also known as the MAPK/ERK) pathways, leads to uncontrolled cell division, differentiation, proliferation, motility, and apoptosis (**Figure 2**). Mounting studies have shown that dysregulated IQGAP expression promotes HCC progression and plays a crucial role in HBV-induced HCC.

### 5.1 IQGAPs Influence Multiple Key Oncogenic Pathways in HCC

#### 5.1.1 Wnt/β-Catenin Signaling Pathway

The canonical Wnt/β-catenin signaling pathway is involved in the maintenance of cellular homeostasis and proliferation (99, 100). In the absence of Wnt, β-catenin is trapped in the cytoplasm by a destructive complex resulting in its ubiquitination and eventual degradation. Furthermore, Wnt binds to the transmembrane receptor Frizzled and low-density lipoprotein receptor-related protein 5/6 (LRP5/6) to trigger phosphorylation and activation of Dishevelled family of proteins. This results in the prevention of β-catenin destruction, leading to its release and accumulation, followed by translocation to the nucleus, where it binds to transcription factors and activates the transcription of downstream target genes and ultimately leads to aberrant cell proliferation and neoplasm formation. Scaffold proteins have been shown to upregulate Wnt/β-catenin signaling in human HCC, thereby contributing to the malignant progression of HCC (101). Interestingly, β-catenin expression and signaling activity in HCC tissues is positively correlated with IQGAP1 expression and inversely correlated with IQGAP2 expression (24, 93). Furthermore, these studies showed upregulation of IQGAP1 and downregulation of IQGAP2 exceptionally activate the Wnt/β-catenin signaling pathway. Increased Wnt/β-catenin signaling is strongly implicated in increased HCC cell proliferation, migration, and EMT, which is clinicopathologically correlated with the degree of tumor malignancy (94). However, the elucidation of the precise mechanisms of how IQGAPs activate the Wnt/β-catenin signaling pathway warrants further study.

#### 5.1.2 Hippo Signaling Pathway

The Hippo signaling pathway is evolutionarily conserved and governs organ development and homeostasis by regulating cell proliferation, apoptosis, cell fate, and stem cell self-renewal (102, 103). The core components of Hippo signaling include kinases MST1/2 and LATS1/2, transcriptional cofactors yes-associated-protein-1 (YAP), and transcriptional coactivator with PDZ-binding motif (TAZ). Upon activation of the Hippo kinase cascade, phosphorylated YAP/TAZ is retained in the cytoplasm and then degraded by ubiquitination. Alternatively, the inactivation of upstream kinases results in YAP/TAZ translocation to the nucleus, where they interact with various transcription factors to induce target gene expression (104). Accordingly, the dysregulated Hippo pathway may be involved in cancer initiation and progression by increasing tumor cell proliferation and migration and inhibiting apoptosis (103, 105).

Delgado et al. and Anakk et al. demonstrated that IQGAP1 is an upstream regulator of the Hippo pathway and promotes HCC by activating YAP (29, 92). IQGAP1 can bind to LATS1 and MST2 through the IQ domain and scaffold the MST2-LATS1 complex. However, it inhibits the kinase activity of MST2 and LATS1 and negatively regulates the MST2-LATS1 pro-apoptotic signal (19). Furthermore, bile acids act as promoters of hepatic tumorigenesis and upregulate the Hippo pathway in an IQGAP1-dependent manner (19, 92). In the absence of IQGAP1, bile acids fail to promote cell proliferation because of the reduced levels of IQGAP1-activated YAP (19, 92). Delgado et al. also reported that IQGAP1 activated the YAP-NUAK family kinase 2 (NUAK2) positive feedback loop to promote HCC cell proliferation and growth and accelerate HCC tumorigenesis and growth in mice (29). The inhibition of NUAK2 was shown to attenuate YAP-dependent cancer cell proliferation and liver tumor growth (106). These results suggest that activation of the Hippo signaling pathway by IQGAP1 is essential for hepatocarcinogenesis and progression and may be a promising therapeutic target for liver cancer.

#### 5.1.3 TGF-β Signaling Pathway

The TGF-β signaling pathway affects cellular homeostasis by influencing cell replication, growth, differentiation, and migration (107, 108). Activated TGF-β binds to TGF-β receptor II (TβRII) and recruits the TβRI receptor to form a receptor complex, followed by phosphorylation and activation of a group of related intracellular proteins Smad2/3. Activated Smad2/3 interacts with Smad4 and forms the Smad2/3/4 complex, which translocates to the nucleus and binds to DNA modulating gene expression. During the initial stages of tumor development, TGF-β can induce cancer cell cycle arrest and apoptosis to exert tumor suppressor effects. As tumors progress and cancer cells become resistant to TGF-β-induced apoptosis, TGF-β converts to a tumor-promoting role to enhance cancer cell proliferation, migration, and EMT. TGF-β is highly expressed in HCC, in which cancer-associated fibroblasts (CAFs) derived from stromal cells or hepatocytes are the principal source of TGF-β (109). TGF-β upregulates the expression of transcription repressor Snail and downregulates E-cadherin in HCC to promote EMT, as well as tumor invasion and metastasis, supporting the role of TGF-β in tumor progression and poor patient prognosis (110).

Shi et al. reported that IQGAP3 overexpression amplified TGF-β activity and promoted its translocation into the nucleus, while the expression levels of phosphorylated Smad2, Smad3, and downstream proteins of the TGF-β signaling pathway were also elevated (22). IQGAP3 was also shown to enhance the invasion and migration of HepG2 and HCCLM3 cells. However, these effects were abolished by silencing or knockdown of IQGAP3, silencing Smad3, or treatment with the TGF-β inhibitor SB431542 indicating the important role of the TGF-β signaling pathway in the promotion of IQGAP3-induced invasion and metastasis in HCC (22).

Although IQGAP1- and IQGAP3-activated TGF-β pathways have been shown to promote HCC onset and progression, in another study IQGAP1-induced activation of the TGF-β pathway plays the opposite role in hepatic metastatic carcinoma whereby myofibroblastic differentiation is inhibited (111). The study showed that in colon and lung cancer liver metastases, IQGAP1 binds to TβRII and suppresses TβRII-mediated signaling to prevent HSC differentiation in the tumor microenvironment and constrains metastatic tumor growth. Moreover, in an experimental liver metastasis model, *Iqgap1^-/-^* mice showed higher levels of TGF-β receptor and HSC activation, which promoted metastatic tumor growth (111). Further investigation into signaling interactions between the TGF-β pathway and IQGAPs will provide further insight into how the TGF-β pathway and IQGAPs contribute to primary HCC and hepatic metastatic carcinoma.

#### 5.1.4 PI3K/AKT/mTOR Signaling Pathway

The PI3K/AKT/mTOR signaling pathway plays an important role in cell cycle regulation and is involved in cell metabolism, proliferation, apoptosis, and carcinogenesis (112, 113). Numerous cell surface RTKs are involved in the pathways, including insulin receptor (InsR), EGFR, platelet-derived growth factor receptor (PDGFR), hepatocyte growth factor receptor (HGFR), discoidin domain receptor (DDR), leukocyte tyrosine kinase (LTK), and others, which have high affinity for numerous polypeptide growth factors, cytokines, and hormones and are activated by them (114). Activated RTKs phosphorylate and activate downstream kinases leading to the activation of AKT. Subsequently, AKT activates mTOR, causing the increased translation of genes involved in angiogenesis and cell cycle progression, thereby inhibiting apoptosis and promoting cell growth and migration. Additionally, activated mTOR complex 2 (mTORC2) can phosphorylate AKT, causing excessive activation of AKT. Dysregulated RTKs activate the PI3K/AKT/mTOR signaling pathway in HCC and amplify tumor progression, metastasis, and invasion (115, 116).

IQGAP1 was shown to promote HCC cell growth and proliferation through the PI3K/AKT signaling pathway and facilitate the binding of mTORC2 and AKT to accelerate AKT phosphorylation on Ser-473 (17, 19). Ablation of IQGAP1 either by knockdown or mutation of IQGAP1, or treatment with the PI3K inhibitor LY294002 in cells overexpressing IQGAP1 slowed HCC cell proliferation (17). mTOR was also recently shown to activate S-adenosylmethionine decarboxylase proenzyme (AMD1), which phosphorylates obesity-associated protein (FTO) and subsequently increases the expression of a number of transcription factors that participate in cancer genesis, progression, and stemness (98, 117). In HCC, the interaction of IQGAP1 with FTO increases the phosphorylation and expression of FTO and enhances the stem cell-like properties of HCC cells (98, 117, 118). In addition, IQGAP3 interacts with protein kinase C δ (PKCδ) to competitively inhibit the interaction between PKCδ and PKCα, freeing and activating PKCα, and triggering PI3K/AKT signaling pathways to enhance HCC cell proliferation (21). Similarly, PI3K/AKT activity is increased by binding of IQGAP1 to Rac1, which activates the Src/FAK pathway and can promote HCC cell migration, invasion, and anoikis resistance (20, 119). These results confirm the important role of the PI3K/AKT signaling pathway in the IQGAP-induced HCC progression.

#### 5.1.5 MAPK/ERK Signaling Pathway

The MAPK/ERK signaling pathway plays an important role in cell growth, proliferation, differentiation, and apoptosis (120). Activated RTKs subsequently activate both the PI3K/AKT/mTOR and MAPK/ERK pathways. RTK converts Ras-GDP to Ras-GTP, leading to Ras activation and subsequent activation of Raf, possibly *via* the Src-family tyrosine kinase. Overall, Raf activation promotes phosphorylation and activation of ERK1/2 and triggers downstream signaling pathways in the cytosol, as well as transcription factors in the nucleus to drive cell proliferation and growth. Aberrant activation of the MAPK/ERK signaling pathway was recently shown to promote tumor growth, migration, invasion, and metastasis while also causing resistance to targeted therapies (121, 122).

IQGAP1 and the Ras gene family have been implicated in HCC induction. In the diethylnitrosamine (DEN)-induced mouse liver cancer model, mRNA expressions of IQGAP1 and the Ras gene family were highly elevated in HCC cells compared to normal hepatocytes, and their expression increased in response to the dosage of DEN (95). However, the mRNA expressions of members of Ras gene family significantly decreased after IQGAP1 silencing, which was accompanied by reduced proliferation and invasion ability of HCC cells (97). In addition, ERK and AKT may be involved in IQGAP1-induced elevation of NF-κB, promoting tumorigenesis, metastasis, and resistance to apoptosis (10, 123, 124). These results suggest that IQGAP1 may activate the MAPK/ERK signaling pathway by upregulating Ras gene family expression to promote hepatocarcinogenesis and progression.

### 5.2 Action Mechanisms of IQGAPs in HBV-Mediated HCC

Chronic HBV infection is etiologically responsible for more than half of all HCC cases, particularly in HBV epidemic regions where the rate can reach up to 80% (13). Compared to the non-infected population, chronic HBV carriers have a 10- to 25-fold increased risk of HCC throughout their lifetime (125). Hepatitis B virus X protein (HBx) has been implicated in HBV-related hepatocarcinogenesis and is considered to be a key oncogenic factor (126–128). HBx transgenic mice developed HCC demonstrating the potential independent carcinogenic effects of HBx (129). Therefore, the specific role of HBx in the development of HCC has attracted widespread interest. Xu et al. reported that HBx upregulates Cdc42 expression and activity. The accumulating active Cdc42 interacts with the GRD domain of IQGAP1 and stimulates the proliferation and inhibition of apoptosis of HCC cells (18). In addition, HBV augmented the association between IQGAP1 and Rac1, leading to increased intracellular levels of reactive oxygen species that subsequently accelerated Src kinase phosphorylation which ultimately activates FAK signaling and promotes anoikis resistance, migration, and invasion of HCC cells (20).

### 5.3 Putative Explanation for Opposite Functions of IQGAP1/3 and 2 in HCC

Given that IQGAPs share a similar domain structure and sequence homology, the paradoxical phenomenon that IQGAP1/3 and 2 play contrasting roles in HCC may be due to their different protein binding partners, regulated signaling pathways, subcellular localization, and diverse tissue expression. The Rho family of small GTPases mainly includes Cdc42, Rac, and Rho, which cycle between GTP-bound active and GDP-bound inactive states and regulate multiple cellular processes (130). For example, active Cdc42 stimulates cell proliferation, adhesion, migration, polarity, and dynamic changes in the cytoskeleton (131). Besides, RhoA and RhoC are oncogenic and associated with cancer cell proliferation, invasion, and metastasis, while RhoB is tumor suppressive and promotes apoptosis (132). IQGAP1/3 appear to bind selectively to active GTP-Cdc42 and GTP-Rac1 through the GRD domain, while IQGAP2 binds indiscriminately to both GTP- and GDP-bound forms (40, 43, 133). Casteel et al. found that IQGAP1 interacts directly with active RhoA and RhoC *via* the GRD domain, but not with RhoB (134). Therefore, the differential predisposed binding of IQGAPs to GTPases may lead to their opposite effects in HCC.

In addition to protein binding partners, IQGAPs also regulate different signaling pathways and have opposite effects on the same signaling pathway (**Figure 2**). IQGAP1 exerts oncogenic effects by promoting PI3K/AKT/mTOR, MAPK/ERK, and Wnt/β-catenin signaling pathways while inhibiting the Hippo signaling pathway (17, 19, 94). IQGAP3 also acts as an oncogenic agent by promoting TGF-β and PI3K/AKT/mTOR signaling pathways, while IQGAP2 suppresses carcinogenesis by inhibiting the Wnt/β-catenin signaling pathway (21, 22, 93). Furthermore, IQGAPs are involved in carcinogenesis by regulating the mitosis of mammalian cancer cells (135). Adachi and coworkers revealed that IQGAP1 was distributed uniformly throughout the cell cortex, and IQGAP3 was specifically localized in the equatorial cortex at anaphase in Hela cells, whereas IQGAP2 was not detected throughout the cell (135). Moreover, the role of IQGAP1/3 in mitosis was further confirmed by suppression of IQGAP1 and IQGAP3 which impaired the localization of anillin and RhoA to the contractile ring and inhibited cytokinesis (135). The foregoing interpretations might lead to distinct roles of IQGAPs in HCC, but the concrete mechanisms still warrant further investigation. More in-depth studies on the molecular structures and mechanisms would contribute to providing precise remedy with IQGAPs as targets for cancer therapy.

## 6 Conclusion

This review described the structures of the three members of the IQGAP family and their expression and diverse roles in different liver diseases and mainly in HCC. The signaling pathways associated with IQGAPs and the functional mechanisms in HCC were also presented. Although IQGAPs are promising therapeutic targets for HCC, some issues need to be addressed. For example, IQGAP1 plays distinct roles in primary HCC and hepatic metastatic carcinoma, as well as the concrete mechanism of inter-regulation between IQGAP families. Furthermore, the oncogenes IQGAP1 and IQGAP3 play critical roles in cytoskeletal remodeling of the liver and bile ducts, and in liver regeneration after liver injury, respectively (136–138). Thus, the inhibition of IQGAP1 and IQGAP3 may disrupt the integrity of the liver and bile ducts and affect liver regeneration after liver injury. Future detailed studies on the mechanistic roles of IQGAPs in the cytoskeleton and HCC could promote more precise targeted therapy for HCC while avoiding disruption of the normal physiological functions of IQGAPs. Likewise, it is equally important to explore the role of these fundamental scaffolding proteins in other liver diseases such as fatty liver disease, fibrosis, and cirrhosis to unravel the functional relationship of these proteins with chronic liver diseases which if left untreated ultimately lead to HCC.

## Author Contributions

QD and QA summarized and wrote the article. MR, FS, and AZ commented and revised the manuscript. All authors contributed to the article and approved the submitted version.

## Funding

This study was supported by funding from the *Friedrich-Schiller-Universität Jena* and Foundation “Else Kröner-Fresenius-Stiftung” within the Else Kröner Graduate School for Medical Students “*Jena School for Ageing Medicine (JSAM)*”.

## Conflict of Interest

The authors declare that the research was conducted in the absence of any commercial or financial relationships that could be construed as a potential conflict of interest.

## Publisher’s Note

All claims expressed in this article are solely those of the authors and do not necessarily represent those of their affiliated organizations, or those of the publisher, the editors and the reviewers. Any product that may be evaluated in this article, or claim that may be made by its manufacturer, is not guaranteed or endorsed by the publisher.
